# Characterization of Aminoglycoside Resistance and Virulence Genes among *Enterococcus spp.* Isolated from a Hospital in China

**DOI:** 10.3390/ijerph120303014

**Published:** 2015-03-11

**Authors:** Wanxiang Li, Jing Li, Quhao Wei, Qingfeng Hu, Xiaowei Lin, Mengquan Chen, Renji Ye, Huoyang Lv

**Affiliations:** 1 Center of Laboratory Medicine, Zhejiang Provincial People’s Hospital, 158 Shangtang Road, Hangzhou 310014, China; E-Mails: lab_lwx@126.com (W.L.); lab_wqh@126.com (Q.W.); lab_hqf@126.com (Q.H.); 2 Medical College, Shihezi University, 280 Beisan Road, Shihezi 832000, China; E-Mail: lj165613686@163.com; 3 Second Clinical Medical College, Zhejiang Chinese Medical University, 548 Binwen Road, Hangzhou 310053, China; E-Mail: lab_lxw@126.com; 4 Wenzhou Hospital of Traditional Chinese Medicine, 27 Dashimen Road Xinhe Street, Wenzhou 325000, China; E-Mail: cmq290340238@126.com; 5 Medical College, Taizhou University, 1139 Shifu Road, Taizhou 318000, China; E-Mail: yerenji11@163.com

**Keywords:** *Enterococcus* species, high-level aminoglycoside resistance (HLAR), virulence genes, multi-drug resistance

## Abstract

This study investigated the aminoglycoside resistance phenotypes and genotypes, as well as the prevalence of virulence genes, in *Enterococcus* species isolated from clinical patients in China. A total of 160 enterococcal isolates from various clinical samples collected from September 2013 to July 2014 were identified to the species level using the VITEK-2 COMPACT system. The antimicrobial susceptibilities of the identified *Enterococcus* strains were determined by the Kirby-Bauer (K-B) disc diffusion method. PCR-based assays were used to detect the aminoglycoside resistance and virulence genes in all enterococcal isolates. Of 160 *Enterococcus* isolates, 105 were identified as *E. faecium*, 35 as *E. faecalis*, and 20 isolates were classified as “other” *Enterococcus* species. High-level aminoglycoside resistance (HLAR) for gentamicin, streptomycin, and both antibiotics was identified in 58.8, 50, and 34.4% of strains, respectively. The most common virulence gene (50.6% of isolates) was *efaA*, followed by *asa1* (28.8%). The most prevalent aminoglycoside resistance genes were *aac(6')-Ie-aph(2'')*, *aph(2')-Id*, *aph(3')-IIIa*, and *ant(6')-Ia*, present in 49.4%, 1.3%, 48.8% and 31.3% of strains, respectively. Overall, *E. faecium* and *E. faecalis* were most frequently associated with hospital-acquired enterococcal infections in Zhejiang Province. All aminoglycoside resistance genes, except *aph*(*2''*)*-Id*, were significantly more prevalent in HLAR strains than amongst high level aminoglycoside susceptible (HLAS) strains, while there was no significant difference between HLAR and HLAS strains in regard to the prevalence of virulence genes, apart from *esp*, therefore, measures should be taken to manage infections caused by multi-drug resistant *Enterococcus* species.

## 1. Introduction

*Enterococcus* species are opportunistic pathogens that are part of the normal gut microbiota of both humans and animals, and can survive in a diverse range of harsh conditions [1,2]. The first *Enterococcus* species to be identified was isolated from an endocarditis patient in the 1900s. Since then, enterococci have been recognized as an important cause of nosocomial infections [3]. Recently, there has been an increase in invasive therapy because of the frequent inappropriate use of antimicrobial agents. In addition, high-level aminoglycoside-resistant (HLAR) enterococci and vancomycin-resistant enterococci (VRE) have caused significant problems for clinical anti-infective therapy. VRE strains are becoming more widespread in North America, Europe, and Asia, although prevalence rates vary greatly among different geographical areas. Six genotypes (*vanA*, *vanB*, *vanC*, *vanD*, *vanE*, and *vanG*) have been described, of which, *vanA* (Tn1546) and *vanB* (Tn1549/Tn5382) are the most common [4]. HLAR strains are becoming more prevalent, mostly because of aminoglycoside modifying enzymes (AMEs) that are encoded within mobile genetic elements, which are widespread amongst enterococci [5]. Among the AMEs, the most prevalent gene is *aac(6')-Ie-aph(2'')*, which encodes a bifunctional enzyme, AAC(6')-APH(2''), that confers resistance to a broad spectrum of aminoglycosides [6]. Other resistance genes, encoding the AMEs 2*'*-O-phosphotransferase (APH(2*'*)), 3*'*-O-phosphotransferase (APH(3*'*)), 3*'*-O-adenyltransferase (ANT(3*'*)), 4-O-adenyltransferase (ANT(4*'*)), and 6*'*-O-adenyltransferase (ANT(6*'*)) are also found on these mobile genetic elements. Enterococci also produce many virulence factors, including collagen-binding protein (*ace*), aggregation substance (*asa1*), cytolysin (*cylA*), endocarditis antigen (*efaA*), enterococcal surface protein (*esp*), gelatinase (*gelE*), and hyaluronidase (*hyl*) [7,8,9].

Generally, *Enterococcus* species are not recognized as dominant pathogens, and usually only cause infections in patients who have a severe underlying disease or are immunocompromised [2]. Penicillin, ampicillin, and aminoglycosides are the first-line drugs for the treatment of enterococcal infection [3]. However, enterococci rapidly acquire antibiotic resistance determinants, as shown by the increasing number of infections caused by HLAR *Enterococcus* species. The aim of present study was to determine the prevalence of HLAR strains, examine their antibiotic susceptibility phenotypes, screen for the presence of genes coding for AMEs (including *aac(6')-Ie-aph(2'')*, *aph(2'')-Ib*, *aph(2'')-Id*, *ant(3'')-I*, *ant(4')-Ia*, *ant(6')-Ia*, and *aph(3')-IIIa*) and other virulence factors (including *ace*, *asa1*, *cylA*, *efaA*, *esp*, *gelE*, and *hyl*), and examine the relationships between AME- and virulence-encoding genes of enterococci isolated from clinical patients in China.

## 2. Materials and Methods

### 2.1. Bacterial Isolation and Identification

This study was performed between September 2013 and July 2014 at Zhejiang Province People’s Hospital, Hangzhou, China, a 2000-bed tertiary hospital. Rectal swab or stool specimens were collected from all patients admitted to the 40-bed ICU, and all VRE strains isolated from other clinical samples collected from June 2011 to January 2014 were also examined. Duplicate isolates were discarded. Each enterococcal isolate was first identified using the VITEK-2 COMPACT fully automated microbiological system (bioMérieux, Inc., Durham, NC, USA). Genotyping was carried out by screening for the presence of aminoglycoside resistance and other virulence genes using a multiplex polymerase chain reaction (PCR) method.

### 2.2. Susceptibility Testing

The antibacterial agents tested were gentamicin (120 μg), streptomycin (300 μg), erythromycin (15 μg), vancomycin (30 μg), teicoplanin (30 μg), ciprofloxacin (5 μg), levofloxacin (5 μg), chloramphenicol (30 μg), quinupristin-dalfopristin (15 μg), penicillin (10 U), ampicillin (10 μg), linezolid (30 μg), tetracycline (30 μg), and rifampin (5 μg). Susceptibility phenotypes were determined using the disc diffusion method according to the CLSI guidelines. *E. faecalis* ATCC29212 was used as a reference strain.

### 2.3. Amplification of Virulence and Resistance Genes

Template DNA was extracted using an EZ-10 spin column bacterial genomic DNA miniprep kit (Bio Basic Inc., Markham, ON, Canada). Genes encoding AMEs, including *aac(6')-Ie-aph(2'')*, *aph(2'')-Ib*, *aph(2'')-Id*, *aph(3')-IIIa*, *ant(3'')-I*, *ant(4')-Ia*, and *ant(6')-Ia*, along with the virulence genes *ace*, *asa1*, *cylA*, *efaA*, *esp*, *gelE*, and *hyl*, were examined by PCR. Detection was performed in a final volume of 20 μL, containing 400 nM of each primer (primer sequences are provided in Table 1), 10 µL of premix Taq polymerase (Takara, Otsu, Japan) containing MgCl_2_, dNTPs, and reaction buffer, 1 µL of DNA template, and dd H_2_O to 20 µL. The PCR conditions consisted of a pre-denaturation step at 94 °C for 4 min, followed by 35 cycles of 94 °C for 40 s, 55 °C for 40 s, and 72 °C for 45 s. A final extension step was performed at 72 °C for 5 min.

**Table 1:** PCR primers used in the amplification of virulence and resistance genes.

Gene	Description	Sequence(5 *'*-3*'*)	Amplicon Size (bp)	Ref.
*ace*	Collagen-binding protein	GGAATGACCGAGAACGATGGC	616	[8]
		GCTTGATGTTGGCCTGCTTCCG		
*asa1*	Aggregation substance	CACGCTATTACGAACTATGA	375	[10]
		TAAGAAAGAACATCACCACGA		
*ylA*	Cytolysin	ACTCGGGGATTGATAGGC	688	[8]
		GCTGCTAAAGCTGCGCTT		
*efaA*	Endocarditis antigen	CGTGAGAAAGAAATGGAGGA	499	[7]
		CTACTAACACGTCACGAATG		
*esp*	Enterococcal surface protein	AGATTTCATCTTTGATTCTTGG	510	[10]
		AATTGATTCTTTAGCATCTGG		
*gelE*	Gelatinase	TATGACAATGCTTTTTGGGAT	213	[10]
		AGATGCACCCGAAATAATATA		
*hyl*	Hyaluronidase	ACAGAAGAGCTGCAGGAAATG	276	[10]
		GACTGACGTCCAAGTTTCCAA		
*aac(6')-Ie-aph(2")-Ia*	AAC(6*'*)-APH(2*''*)	AGGAATTTATCGAAAATGGTAGAAAAG	369	[6]
		CACAATCGACTAAAGAGTACCAATC		
*aph(3')-IIIa*	APH(3*'*)	GGCTAAAATGAGAATATCACCGG	523	[6]
		CTTTAAAAAATCATACAGCTCGCG		
*ant(4')-Ia*	ANT(4*'*)	CAAACTGCTAAATCGGTAGAAGCC	294	[6]
		GGAAAGTTGACCAGACATTACGAACT		
*aph(2")-Ic*	APH(2*'*)	CCACAATGATAATGACTCAGTTCCC	444	[11]
		CCACAGCTTCCGATAGCAAGAG		
*aph(2")-Ib*	APH(2*'*)	CTTGGACGCTGAGATATATGAGCAC	867	[12]
		GTTTGTAGCAATTCAGAAACACCCTT		
*aph(2")-Id*	APH(2*'*)	GGTGGTTTTTACAGGAATGCCATC	642	[11]
		CCCTCTTCATACCAATCCATATAACC		
*ant(3")-III*	ANT(3*'*)	CACGCTATTACGAACTATGA	284	[11]
		TAAGAAAGAACATCACCACGA		
*ant(6')-Ia*	ANT(6*'*)	ACTCGGGGATTGATAGGC	597	[11]
		GCTGCTAAAGCTGCGCTT		

The amplified PCR products were analyzed on 1% (*w/v*) agarose gels. DNA bands were visualized by staining with ethidium bromide and photographed under UV illumination.

### 2.4. Statistical Analyses

VRE data was managed using the WHONET version 5.6 software (The WHONET Team, Boston, MA, USA). SPSS software version 17.0 (IBM, Inc., Armonk, NY, USA) was used for statistical analyses. Differences in antimicrobial sensitivity profiles and the prevalence of resistance and virulence genes between HLAR and HLAS *Enterococcus* species were compared using the Chi-square test, with a *p*-value < 0.05 indicating statistical significance.

## 3. Results

### 3.1. Antimicrobial Susceptibility

Of the 160 *Enterococcus* isolates, 105 were identified as *E. faecium*, 35 as *E. faecalis*, and 20 as “*other*” *Enterococcus* species. The antimicrobial susceptibility profiles of all of the isolates are shown in Table 2. HLAR for gentamicin, streptomycin, and both antibiotics was identified in 58.8%, 50%, and 34.4% of isolates, respectively. In the present study, resistance to either gentamicin or streptomycin (or both) was regarded as indicating HLAR. In total, 119 (74.4%) of the isolates were classified as HLAR. In contrast, very little resistance to linezolid or teicoplanin was detected amongst the 160 isolates. Amongst the HLAR strains, comparison of the two species showed that rates of resistance were significantly higher in *E. faecium* than in *E. faecalis* for all antibiotics examined, except ERY, LZD, and CHL. Among the HLAS strains, a significant difference was again observed for all antibiotics, except TET, CHL, TEC, and VAN. Comparison of HLAR vs. HLAS *E. faecium* isolates revealed that rates of antibiotic resistance were only significantly different for TEC and VAN, while a significant difference was only observed for erythromycin between the HLAR and HLAS *E. faecalis* isolates.

### 3.2. Distribution of Virulence Genes

The prevalence of the *ace*, *asa1*, *cylA*, *efaA*, *esp*, *gelE*, and *hyl* virulence genes amongst the 160 *Enterococcus* species was 15%, 28.8%, 19.4%, 50.6%, 21.9%, 20.6%, and 19.6%, respectively. In bivariate analysis, the overall rate of virulence gene carriage amongst the *E. faecalis* group was significantly higher (*n* = 35) than in the *E. faecium* group (*n* = 105) or the “*other*” group (*n* = 20) (except *hyl* and *esp*). *ace* was significantly (*p* < 0.001) more prevalent amongst the *E. faecalis* strains (*n* = 35, 60%) than the *E. faecium* strains (*n* = 105, 1.5%) or the “*other*” strains (*n* = 20, 5%), as were *cylA* (*p* < 0.001), *gelE* (*p* < 0.001), *efaA* (*p* < 0.001), and *asa1* (*p* < 0.001). There was no significant difference between *E. faecalis* and *E. faecium* with regard to *esp* (*E. faecalis vs. E. faecium*, *p* = 0.377), although *esp* was significantly more prevalent in both species than in the “*other*” *Enterococcus* spp. group (*E. faecalis vs.* “*other*” strains, *p* = 0.036; *E. faecium vs.* “*other*” strains, *p* = 0.002).

**Table 2:** Antibiotic susceptibility profiles of the tested *Enterococcus* species.

Anti-Microbial Agent	HLAR (119)									HLAS (41)									*P_1_*	*P_2_*	*P_3_*	*P_4_*
	*E.faecium* (82)			*E.faecalis* (27)			*Others* (10)			*E.faecium* (23)			*E.faecalis* (8)			*Others* (10)						
	R	I	S	R	I	S	R	I	S	R	I	S	R	I	S	R	I	S				
AMP ^*^	80 (97.6)	0 (0)	2 (2.4)	1 (3.7)	0 (0)	26 (96.3)	2 (20)	0 (0)	8 (80)	22 (95.7)	0 (0)	1 (4.3)	0 (0)	0 (0)	8 (100)	3 (30)	0 (0)	7 (70)	0.00	0.00	1.00	1.00
G ^*^	80 (97.6)	0 (0)	2 (2.4)	1 (3.7)	0 (0)	26 (96.3)	2 (20)	0 (0)	8 (80)	22 (95.7)	0 (0)	1 (4.3)	0 (0)	0 (0)	8 (100)	1 (10)	0 (0)	9 (90)	0.00	0.00	1.00	1.00
LEF ^*^	80 (97.6)	0 (0)	2 (2.4)	15 (55.6)	0 (0)	12 (44.4)	7 (70)	0 (0)	3 (30)	23 (100)	0 (0)	0 (0)	2 (25)	0 (0)	6 (75)	3 (30)	0 (0)	7 (70)	0.00	0.00	1.00	0.26
ERY ^*^	80 (97.6)	0 (0)	2 (2.4)	23 (85.2)	3 (11.1)	1 (3.7)	9 (90)	0 (0)	1 (10)	18 (78.3)	4 (17.4)	1 (4.3)	2 (25)	3 (37.5)	3 (37.5)	8 (80)	1 (10)	1 (10)	1.00	0.07	1.00	0.04
CIP ^*^	80 (97.6)	0 (0)	2 (2.4)	15 (55.6)	4 (14.8)	8 (29.6)	8 (80)	1 (10)	1 (10)	23 (100)	0 (0)	0 (0)	1 (12.5)	2 (25)	5 (62.5)	3 (30)	2 (20)	5 (50)	0.00	0.00	1.00	0.20
LZD ^*^	3 (3.7)	0 (0)	79 (96.3)	0 (0)	0 (0)	27 (100)	0 (0)	0 (0)	10 (100)	0 (0)	0 (0)	23 (100)	0 (0)	0 (0)	8 (100)	0 (0)	0 (0)	10 (100)	0.57	--	0.82	--
Q/D ^*^	8 (9.8)	6 (7.3)	68 (82.9)	--	--	--	7 (70)	2 (20)	1 (10)	3 (13.0)	2 (8.7)	18 (78.3)	--	--	--	5 (50)	2 (20)	3 (30)	--	--	0.84	--
TET ^*^	36 (43.9)	1 (1.2)	45 (54.9)	20 (74.1)	1 (3.7)	6 (22.2)	10 (100)	0 (0)	0 (0)	12 (52.2)	0 (0)	11 (47.8)	4 (50)	0 (0)	4 (50)	8 (80)	0 (0)	2 (20)	0.00	1.00	0.55	0.27
RIF ^*^	64 (78.1)	7 (8.5)	11 (13.4)	8 (29.6)	5 (18.5)	14 (51.9)	1 (10)	0 (0)	9 (90)	16 (69.6)	5 (21.7)	2 (8.7)	1 (12.5)	1 (12.5)	6 (75)	3 (30)	0 (0)	7 (70)	0.00	0.00	0.80	0.45
CHL ^*^	4 (4.9)	3 (3.7)	75 (91.5)	2 (7.4)	0 (0)	25 (92.6)	0 (0)	1 (10)	9 (90)	1 (4.3)	1 (4.3)	21 (91.4)	1 (12.5)	1 (12.5)	6 (75)	0 (0)	0 (0)	10 (100)	1.00	0.57	1.00	0.46
TEC ^*^	15 (18.3)	18 (22.0)	49 (59.7)	0 (0)	1 (3.7)	26 (96.3)	0 (0)	0 (0)	10 (100)	1 (4.3)	1 (4.3)	21 (91.4)	1 (12.5)	0 (0)	7 (87.5)	0 (0)	0 (0)	10 (100)	0.00	1.00	0.00	0.94
VAN ^*^	43 (52.4)	0 (0)	39 (47.6)	2 (7.4)	1 (3.7)	24 (88.9)	0 (0)	0 (0)	10 (100)	2 (8.7)	0 (0)	21 (91.3)	1 (12.5)	0 (0)	7 (87.5)	0 (0)	0 (0)	10 (100)	0.00	1.00	0.00	1.00

^*^ AMP (ampicillin); G (penicillins); LEF (levofloxacin); ERY (erythromycin); CIP (ciprofloxacin); LZD (linezolid); Q/D (quinupristin-dalfopristin); TET (tetracycline); RIF (rifampin); CHL (chloramphenicol); TEC (teicoplanin); VAN (vancomycin). *P_1_* In HLAR strains compares *E.faecium with E.faecalis. P_2_ In HLAS* compare *E.faecium with E.faecalis. P_3_* Compare HLAR *E.faecium with HLAS E.faecium. P_4_* Compare HLAR *E.faecalis with HLAS E.faecalis.*

*hyl* was significantly more prevalent in *E. faecium* and “*other*” strains than in *E. faecalis* (*E. faecalis* vs. *E. faecium*, *p* < 0.001; *E. faecalis vs.* other strains, *p* = 0.027), but there was no significant difference between *E. faecium* and “*other*” strains (*p* = 0.089).

### 3.3. Distribution of Aminoglycoside Resistance Genes

All the *Enterococcus* species were tested for the presence of aminoglycoside resistance genes. *aac(6')-Ie-aph(2'')*, *aph(2')-Id*, *aph(3')-IIIa*, and *ant(6')-Ia* were found in 49.4%, 1.3%, 48.8%, and 31.3% of isolates, respectively. The distribution of aminoglycoside resistance genes according to phenotype is presented in Table 3. Neither *ant(3'')-I* nor *aph(2')-Ib* were detected among the HLAR isolates. The most prevalent resistance gene was *aph(3')-IIIa* (*n* = 66 isolates), followed by *aac(6')-Ie-aph(2'')* (*n* = 65). Overall, 10 isolates contained *aph(3')-IIIa*, 22 had *aac(6')-Ie-aph(2'')*, seven carried both *aac(6')-Ie-aph(2'')* and *aph(3')-IIIa*, 32 harbored *aph(3')-IIIa*, *ant(6')-Ia*, and *aac(6')-Ie-aph(2'')*, and 15 had *aph(3')-IIIa* and *ant(6')-Ia*. Only two isolates had both *aac(6')-Ie-aph(2'')* and *ant(6')-Ia*, while two others had *aac(6')-Ie-aph(2'')*, *aph(2'')-Id*, and *aph(3')-IIIa*. However, among the HLAS isolates, the most prevalent resistance gene was *aac(6')-Ie-aph(2'')* (*n* = 14), followed by *aph(3')-IIIa* (*n* = 12). *ant(6')-Ia* was only was detected in one isolate. Chi-square tests showed that several resistance genes were significantly more prevalent in HLAR strains than HLAS strains, including *aac(6')-Ie-aph(2'')* (*p =* 0.024), *aph(3')-IIIa* (*p* = 0.004), and *ant(6')-Ia* (*p* < 0.001). In bivariate analysis, *aac(6')-Ie-aph(2'')* was significantly more prevalent in *E. faecium* than in *E. faecalis* and “*other*” strains (*p* < 0.001 in both cases). *aph(3')-IIIa* and *ant(6')-Ia* were significantly more prevalent in *E. faecalis* than in “other” strains (*p* = 0.001 and *p* = 0.024, respectively).

### 3.4. Correlation between Aminoglycoside Resistance Genes and Virulence Genes

**Figure 1:**
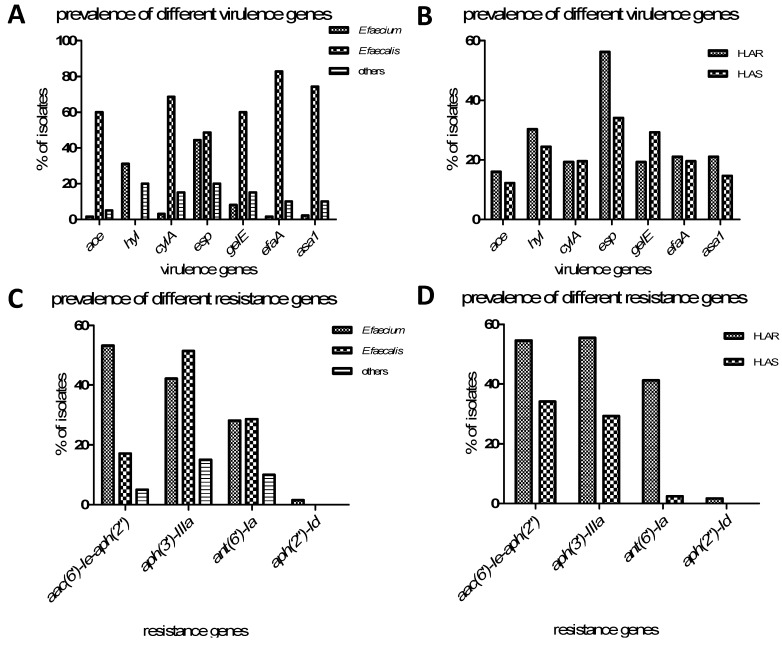
Frequency of different virulence and resistance genes in *Enterococcus* species.

Of the 82 *esp*-positive isolates, the majority (*n* = 66, 80.5%) were resistant to aminoglycosides (Table 3). However, only 52 of the 78 (66.7%) *esp*-negative isolates were resistant to high-level aminoglycosides. Bivariate analysis confirmed that this difference between the *esp*-positive and negative isolates was significant (*p* = 0.047). Of the 119 HLAR isolates, 67 (56.3%) were *esp*-positive; however, only 14 of the 41 (34.1%) HLAS isolates were *esp*-positive (*p* = 0.014) (Figure 1).

## 4. Discussion

In recent years, infections caused by multidrug-resistant Gram-positive organisms have become a significant cause of morbidity and mortality [13,14]. Among these Gram-positive organisms, *Enterococcus* species are the second most common nosocomial bloodstream pathogens isolated in the United States [15,16]. *Enterococci* have been considered nosocomial pathogens since the early 1970s, and high-level resistance to aminoglycosides has become a serious problem in most healthcare facilities [17]. Schouten, *et al.* [18] reported that high level resistance to gentamicin has been detected in all investigated European countries, with prevalence ranging from 1% to 48% (mean, 22.6% ± 12.3%) in 1997. Moreover, there were no geographic relationships among the studied countries. However, in the present study, HLAR was detected in 119 (74.4%) of the isolates, which is significantly higher than previously reported.

**Table 3:** Frequency of different virulence genes in *Enterococcus* species.

No.of Isolates	Phenotype	Genotype				No.of Virulence Genes						
		*aac(6')-Ie-aph(2'')*	*aph(2'')-Id*	*aph(3')-IIIa*	*ant(6')-Ia*	*ace*	*hyl*	*cylA*	*esp*	*gelE*	*efaA*	*asa1*
22	R*	+				4	9	1	12	5	4	3
10	R			+		2	2	5	7	3	4	4
7	R	+		+		1	4	0	5	1	1	1
2	R	+			+	0	0	0	1	0	0	0
15	R			+	+	7	4	7	6	5	7	7
2	R	+	+	+		0	2	0	0	0	0	0
32	R	+		+	+	1	13	1	28	4	2	3
29	R					4	2	9	8	5	7	7
total	119					19	36	23	67	23	25	25
9	S ^*^	+				0	3	0	5	2	0	0
6	S			+		1	2	3	2	1	2	1
5	S	+		+		0	2	2	0	2	1	1
1	S			+	+	1	0	0	1	0	1	1
20	S					3	3	3	6	7	4	3
total	41					5	10	8	14	12	8	6

Notes: + = positive. ^*^ R (resistance); S (susceptible).

It appears that since the 1970s, the prevalence of HLAR species has rapidly increased. In the current study, a significantly higher prevalence of resistance to ampicillin, vancomycin, rifampicin, penicillin, teicoplanin, ciprofloxacin, and levofloxacin was detected in *E. faecium* than in *E. faecalis* (*p* < 0.05), while a greater prevalence of resistance to chloramphenicol, minocycline, and tetracycline was found in *E. faecalis* than in *E. faecium* (*p* < 0.05).

This study also investigated the prevalence of aminoglycoside resistance genes among the 160 *Enterococcus* isolates. In total, 119/160 (74.4%) isolates showed a HLAR phenotype. A previous study by Padmasini *et al*. found that all HLAR *E. faecalis* and *E. faecium* isolates included in their study carried *aac(6')-Ie-aph(2'')-Ia* [17,19]. However, in our study, only 65 (54.6%) of the 119 HLAR strains identified by the Kirby-Bauer method carried *aac(6')-Ie-aph(2'')-Ia*. Interestingly, 29/119 strains (24.4%) did not carry any of the examined aminoglycoside resistance genes, which was lower than has been reported previously [6,17]. While previous studies found that *aac(6')-Ie-aph(2'')-Ia* was the most common aminoglycoside resistance gene, in this study, the most prevalent gene was *aph(3')-IIIa*, which was detected in 55.5% (66/119) of isolates. In a previous studies Padmasini *et al*, 20.2% (39/178) of strains carried both *aac(6')-Ie-aph(2'')-Ia* and *aph(3')-IIIa*, and Emaneini, *et al*. [6] reported that 35.9% of *Enterococcus* isolates examined in their study contained both genes. In our study, *aac(6')-Ie-aph(2'')-Ia* and *aph(3')-IIIa* coexisted in 28.8% of the *Enterococcus* species isolates, which was similar to previous results.

Previous studies by Creti, *et al.* [8] and Billström, *et al*. [10] showed that the virulence genes *ace*, *asa1*, *cylA*, *efaA*, *esp*, *gelE*, and *hyl* are present at varying levels in *E. faecalis* isolates. In the current study, *esp* was the most frequently identified virulence gene (50.6% of isolates), followed by *hyl* (28.8%). These findings differed from the results of Zou *et al*., who showed that *gelE* was the most prevalent virulence gene, and that *hyl* and *cylA* were not detected, while Vankerckhoven, *et al*. [20] detected *esp* in 65% of isolates, and *gelE* and *cylA* were not detected. Comparison of virulence genes amongst HLAR and HLAS strains in the current study showed that only *esp* was significantly more prevalent in HLAR isolates than in HLAS isolates. Other than *hyl* and *esp*, all tested virulence genes, including *ace*, *cylA*, *efaA*, *gelE*, and *asa1*, were significantly more prevalent in *E. faecalis* compared with *E. faecium.* These differences indicate that virulence genes are present at different levels between human and animal isolates [9], and that with the passage of time, *Enterococcus* species have acquired an increasing number of virulence genes.

## 5. Conclusions

In summary, enterococci have become a significant cause of hospital-acquired infections. Therefore, measures should be taken to manage infection caused by multidrug resistant *Enterococcus* species. More and more focus is being placed on mechanisms by many health care facilities to prevent such infections [16,21]. There are some programs to control such infections, including better stewardship of antimicrobial agents and better awareness of the source for pathogen transmission is hospital environment, and health care societies should be widely endorsed [16,22].
